# Transplantation of MITO cells, mitochondria activated cardiac progenitor cells, to the ischemic myocardium of mouse enhances the therapeutic effect

**DOI:** 10.1038/s41598-022-08583-5

**Published:** 2022-03-22

**Authors:** Daisuke Sasaki, Jiro Abe, Atsuhito Takeda, Hideyoshi Harashima, Yuma Yamada

**Affiliations:** 1 Department of Pediatrics, Graduate School of Medicine, Hokkaido University, Kita-15, Nishi 7, Kita-ku, Sapporo, 060-8638 Japan; 2 MRC Mitochondrial Biology Unit, University of Cambridge, Cambridge, UK; 3 Faculty of Pharmaceutical Sciences, Hokkaido University, Kita 12, Nishi 6, Kita-ku, Sapporo, 060-0812 Japan

**Keywords:** Nanobiotechnology, Nanomedicine, Cardiology, Drug delivery, Mitochondria

## Abstract

Given the potential for myocardial stem cell transplantation as a promising treatment for heart failure, numerous clinical trials have been conducted and its usefulness has been clearly confirmed. However, the low rate of engraftment of transplanted cells has become a clinical problem, and this needs to be improved in the case of transplanting cells to the heart. To address this issue, we report on attempts to prepare mitochondria-activated stem cells (MITO cells) for use in transplantation. MITO cells, which is cardiac progenitor cells (CPCs) activated by the mitochondrial delivery of resveratrol with an anti-oxidant and mitochondrial activation effects were successfully prepared using a mitochondrial targeting nanocarrier (MITO-Porter). The purpose of this study was to validate the therapeutic effect of cell transplantation by the MITO cells using a mouse model of myocardial ischemia–reperfusion. Mouse CPCs were used as transplanted cells. The transplantation of CPCs and MITO cells were conducted after myocardial ischemia–reperfusion, and the therapeutic effect was determined. The MITO cells transplanted group showed increase in postoperative weight gain, improve cardiac function and inhibition of fibrosis compared to the non-transplanted group and the CPC group. The transplantation of MITO cells to the ischemic myocardium showed a stronger transplantation effect compared to conventional CPC transplantation.

## Introduction

The incidence of heart failure, mainly due to ischemic heart disease, is on the rise with the aging of the population and the increasing prevalence of hypertension and diabetes mellitus associated with changing lifestyles^1^. While the range of treatment options for heart failure has expanded in recent years, due, in part, to remarkable advances in drug therapy, there is still no effective treatment for heart failure. Surgical treatment, such as heart transplantation, is used in the treatment of severe heart failure, but there are limited indications and a lack of donors^2^. Given this background, a number of clinical trials have been conducted on stem cell transplantation as a possible treatment for heart failure such as ischemic heart disease, dilated cardiomyopathy and congenital heart disease^3–5^. There are, however, many challenges that need to be overcome for cardiac cell transplantation therapy to be successfully achieved. These include inducing the differentiation of transplanted cells into homogeneous cardiomyocytes, the development of arrhythmia after transplantation, the choice of transplantation method, optimization of the timing of therapeutic interventions in terms of the severity of the disease, and the maintenance of sustained cardiac stem cell transplantation effects^6,7^.

Heart failure caused by myocardial infarction is considered to be a mitochondrial disorder associated with ischemia^8^. The lack of an energy supply to intracellular mitochondria that is associated with ischemia and the decreased cellular function associated with the increased production of oxidative stress (ROS) in the mitochondria can cause a decrease in cardiac function^9^. Therefore, strengthening mitochondrial function represents a strategy that might reduce the level of oxidative stress on the heart caused by myocardial infarction and thus reduce the incidence of heart failure, which would contribute to an improvement in cardiac function. We hypothesized that enhancing the mitochondrial function of the transplanted cells themselves could improve the efficiency of transplantation in cell transplantation therapy for heart failure, and furthermore, that the activated mitochondria would improve the chances of surviving heart failure.

In previous studies, we reported on the development of a mitochondria-targeted drug delivery system (DDS) called a MITO-Porter that can contain various types of encapsulated molecules, ranging from small molecules to large molecules such as DNA^10–14^, and we conducted cell and animal experiments using the MITO-Porter system^15,16^. In a previous study, we applied the MITO-Porter technology to the preparation of an S2 peptide-modified MITO-Porter that had resveratrol with antioxidant effects encapsulated within it (MITO-Porter (RES)). The S2 peptide^17^ is similar to the SS peptide^18^, which can reach the mitochondrial inner membrane and preserve electron transport components after being taken up by cells. This was used to generate resveratrol-delivered mitochondria-enhanced cardiac progenitor cells (CPC), referred to as MITO cells^19^. A model mouse of doxorubicin (DOX)-cardiomyopathy, a model of a drug-induced cardiac insufficiency, was prepared, and the MITO cell were transplanted to the myocardia of the mice and their protective effect evaluated^19^.

The purpose of this study was to validate the therapeutic effect of cell transplantation by the MITO cell using a mouse model of myocardial ischemia–reperfusion. In this study, we employed the dual ligand system, in which the surface of MITO-Porter was modified with both the S2 peptide and a mitochondrial RNA aptamer including an RNase P (RP) aptamer, increasing cellular uptake into the CPC compared to that for the conventional MITO-Porter. In this experiment, the transplantation of CPCs and MITO cell into the heart of the mice were conducted after myocardial ischemia/reperfusion, and the therapeutic effect was determined 30 days later. Cardiac functions were evaluated by echocardiography and the determination of the extent of formation of histological fibrosis.

## Results

### Experimental protocol for evaluating the therapeutic effect after transplantation of MITO cells

**Figure 1:**
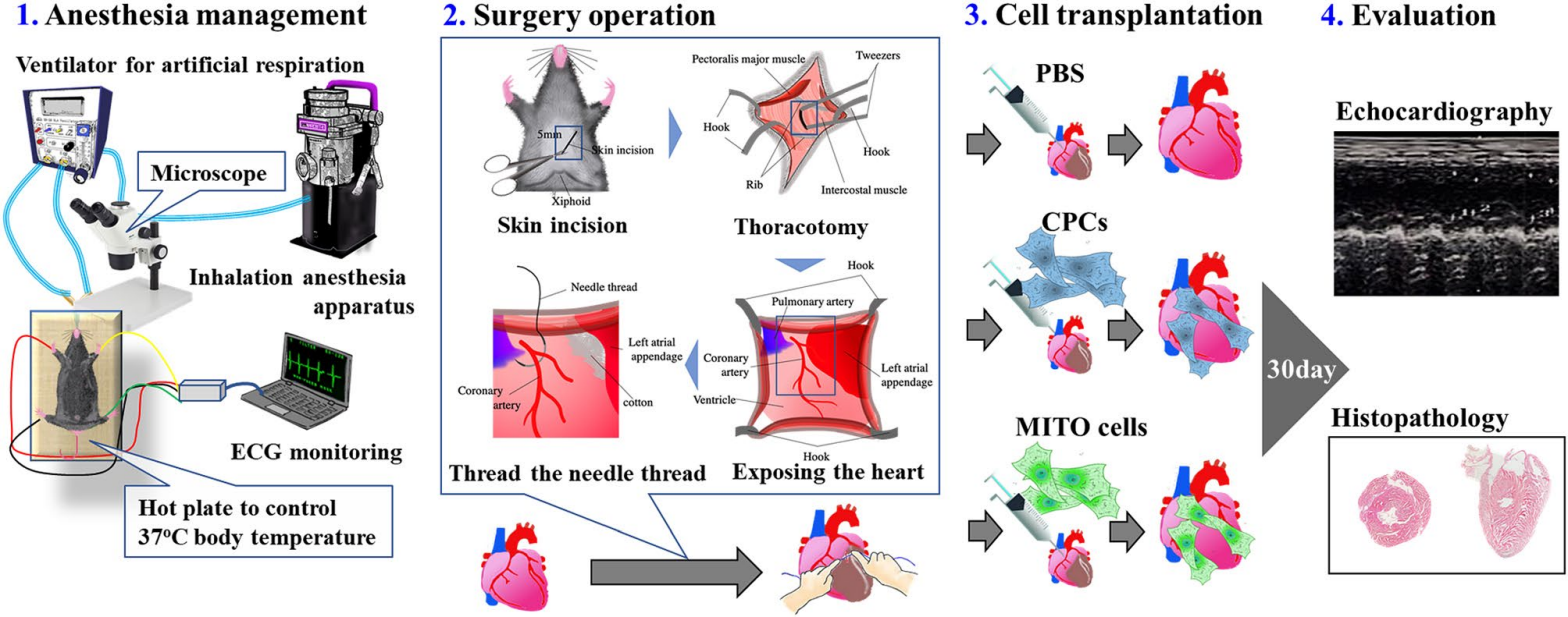
Experimental protocol for evaluating the therapeutic effect with respect to the ischemic cardiomyopathy mouse model after the transplantation of MITO cells. Evaluation of treatment of heart failure by transplanting cells to an ischemic lesion of ischemic cardiomyopathy mouse model (see the Movies 1, 2 for the operation).

In this study, we prepared mitochondria-activated CPCs (MITO cells) from mouse-derived CPCs, and transplanted them into the ischemic myocardium of ischemia–reperfusion model mice to compare the therapeutic effects of this procedure with that for conventional myocardial stem cell transplantation therapy (Fig. 1). We cultured CPCs obtained from mice as previously reported^19^, and the resulting cells were positive for Sca-1, a mesenchymal stem cell marker, confirming that the cells were stem cells as shown in Figure S1. Resveratrol was delivered to mitochondria of the CPCs using the MITO-Porter system to produce MITO cells (Fig. S2).

### Preparation of RP/S2-MITO-Porter (RES)

**Figure 2:**
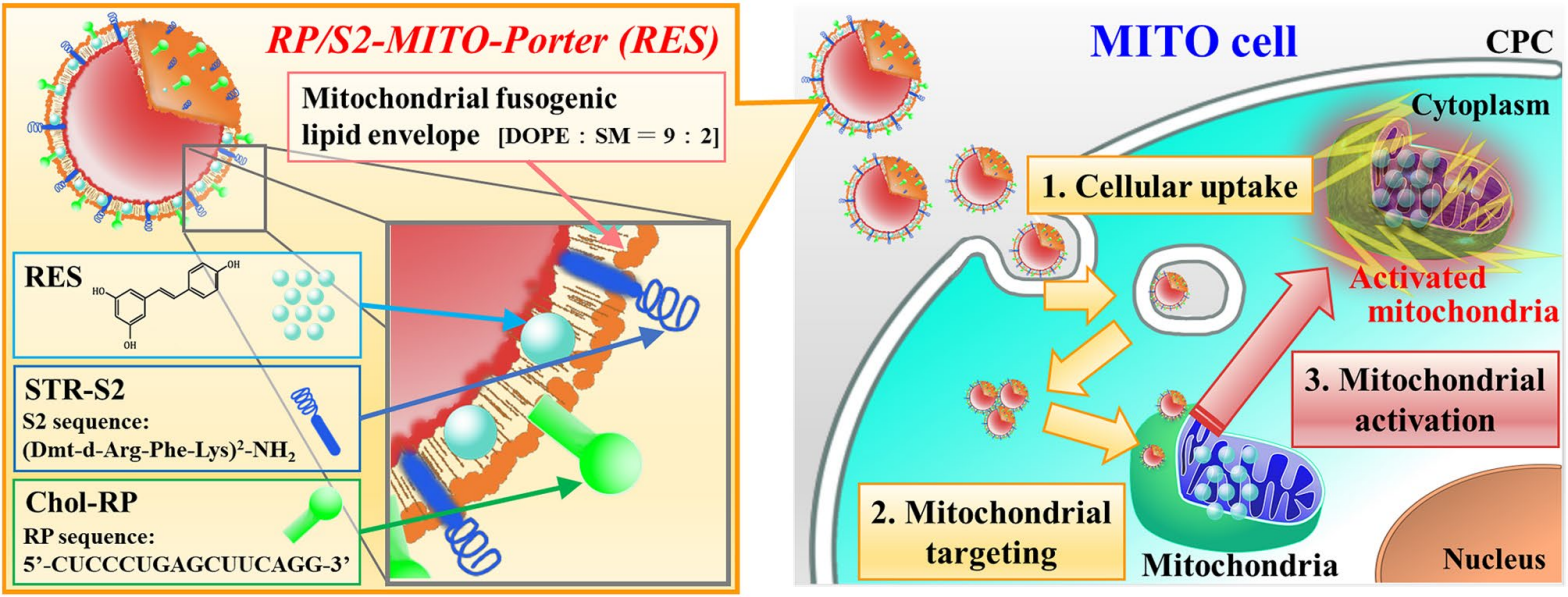
Schematic image of mitochondrial activation in CPC by RP/S2-MITO-Porter. The MITO-Porter (S2 peptide & RP aptamer dual ligand system) could be taken up by the cells (1st step), followed by mitochondrial targeting and a membrane fusion process with mitochondria membrane (2nd step). Finally, mitochondria are activated by mitochondrial delivery of RES to construct MITO-cells. *Chol-RP* cholesteryl RP aptamer, *DOPE* dioleoyl-sn-glycero-3-phosphatidyl ethanolamine, *RES* resveratrol, *SM* sphingomyelin, *STR-S2* stearylated S2 peptide.

We prepared an RP/S2-MITO-Porter (RES) which contained encapsulated resveratrol to activate the mitochondria of the CPCs. The RP/S2-MITO-Porter (RES) is composed of a mitochondrial membrane a highly coalesced lipid bilayer, and resveratrol is encapsulated within the lipid bilayer. The surface of the carriers was modified with the S2 peptide and the RP aptamer. The structure of the RP/S2-MITO-Porter (RES) and a schematic image of its cellular uptake and mitochondrial delivery transport are shown in Fig. 2.

The particle sizes of the prepared carriers were 126 ± 2 nm for the unmodified carrier [DOPE/SM-LP (RES)], 107 ± 11 nm for those containing only S2 peptide [S2-MITO-Porter (RES)] and 129 ± 4 nm for those containing both the S2 peptide and the RP aptamer [RP/S2-MITO-Porter (RES)]. The surface potentials were negative for the DOPE/SM-LP (RES) and RP/S2-MITO-Porter (RES), and positive for the S2-MITO-Porter (RES) (Table 1).

**Table 1:** Particle properties of the carriers encapsulating RES used in this study.

Carrier type	Liposome composition (molar ratio)	Diameter (nm)	Polydispersity index	ζ-potential (mV)
RP/S2-MITO-Porter (RES)	DOPE:SM:STR-S2:Chol-RP:Resveratorl (9:2:1.1:0.1:2)	126 ± 2	0.15 ± 0.05	− 45 ± 2
S2-MITO-Porter (RES)	DOPE:SM:STR-S2:Resveratorl (9:2:1.1:2)	107 ± 11	0.26 ± 0.01	+ 34 ± 3
DOPE/SM-LP (RES)	DOPE:SM (9:2)	129 ± 4	0.13 ± 0.04	− 16 ± 3

Size, PDI, and ζ potential of each particles were measured. *DOPE* 1,2-dioleoyl-*sn*-glycerol-3-phosphatidyl ethanolamine, *Chol-RP* cholesteryl RP aptamer, *SM* sphingomyelin, *STR-S2* stearylated S2 Peptide. S2 peptide sequence: (Dimethyl-Tyr)-(*D*-Arg)-(Phe)-(Lys)-(Dimethyl-Tyr)-(*D*-Arg)-(Phe)-(Lys)-NH_2_. RP aptamer sequence: 5′-UCUCCCUGAGCUUCAGG-3′. Data are represented as mean with S.D. (n = 7).

### Intracellular observation of MITO-Porter (RES)

**Figure 3:**
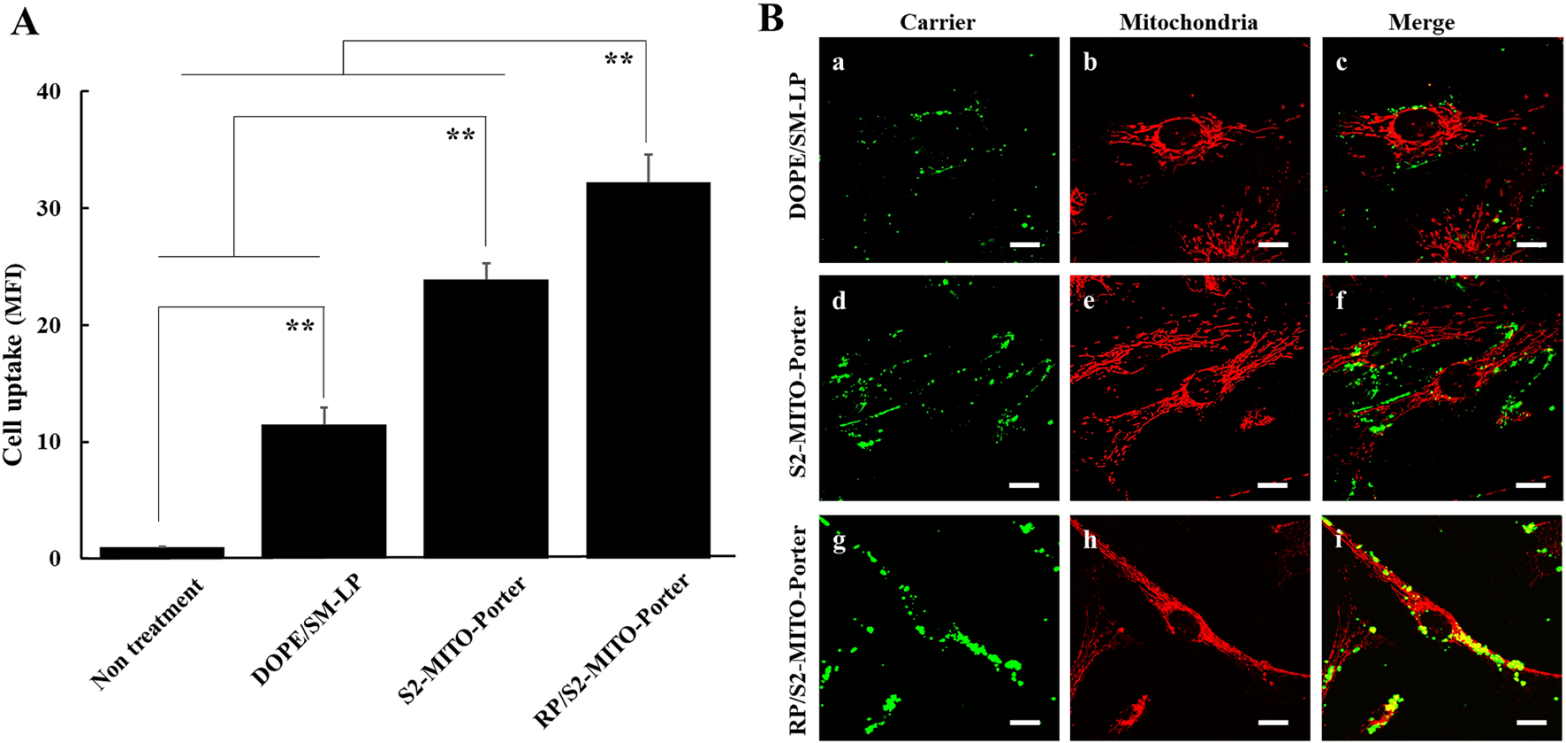
Intracellular observation of MITO-Porter (RES). (**A**) Cellular uptake of RP/S2-MITO-Porter (RES), S2-MITO-Porter (RES) and DOPE/SM-LP (RES) labeled with NBD-lipids were evaluated by flow cytometry, using CPCs. The quantitative analysis of cellular uptake using the mean fluorescence intensity (MFI) of the carriers. Data are represented as the mean with S.D. (n = 3). The significant differences were calculated using one-way ANOVA followed by SNK test (**p < 0.01). (**B**) Intracellular observation of DOPE/SM-LP (RES) (**a**–**c**), S2-MITO-Porter (RES) (**d**–**f**) and RP/S2-MITO-Porter (RES) (**g**–**i**) using CLSM. When green labeled carriers (green color) were co-localized with red stained mitochondria, yellow signals were observed in the merged image. Lines indicate the edges of the cells. Scale bars 20 μm.

We evaluated the cellular uptake of the MITO-Porter (RES) labeled with NBD (fluorescent dye) into the CPC by flow cytometry (Fig. 3A). Compared with DOPE/SM-LP (RES), the uptake of the S2-MITO-Porter (RES) and the RP/S2-MITO-Porter (RES) were significantly increased. There was also a significant difference of the cellular uptake between the S2-MITO-Porter (RES) and the RP/S2-MITO-Porter (RES).

In order to evaluate the intracellular localization of the carriers, we used CLSM to examine the intracellular dynamics of carriers labelled with NBD-DOPE and stained mitochondria red with Mito-Tracker deep red (Fig. 3B). Compared with DOPE/SM-LP (RES), the cellular uptake of the S2-MITO-Porter (RES) and RP/S2-MITO-Porter (RES) was more pronounced with the green dots clearly observed in the cells, indicating that both carriers have cellular uptake ability. It was also observed that red stained mitochondria and green labelled RP/S2-MITO-Porter (RES) were co-localized as yellow signals (Fig. 3Bg–i), while no yellow signals were observed in the case of the use of the S2-MITO-Porter (RES). This co-localization suggests that some of the RP/S2-MITO-Porter (RES) had entered the cytosol and then accumulated in mitochondria.

### Evaluation of the mitochondrial function of MITO cells by extracellular flux analyzer

**Figure 4:**
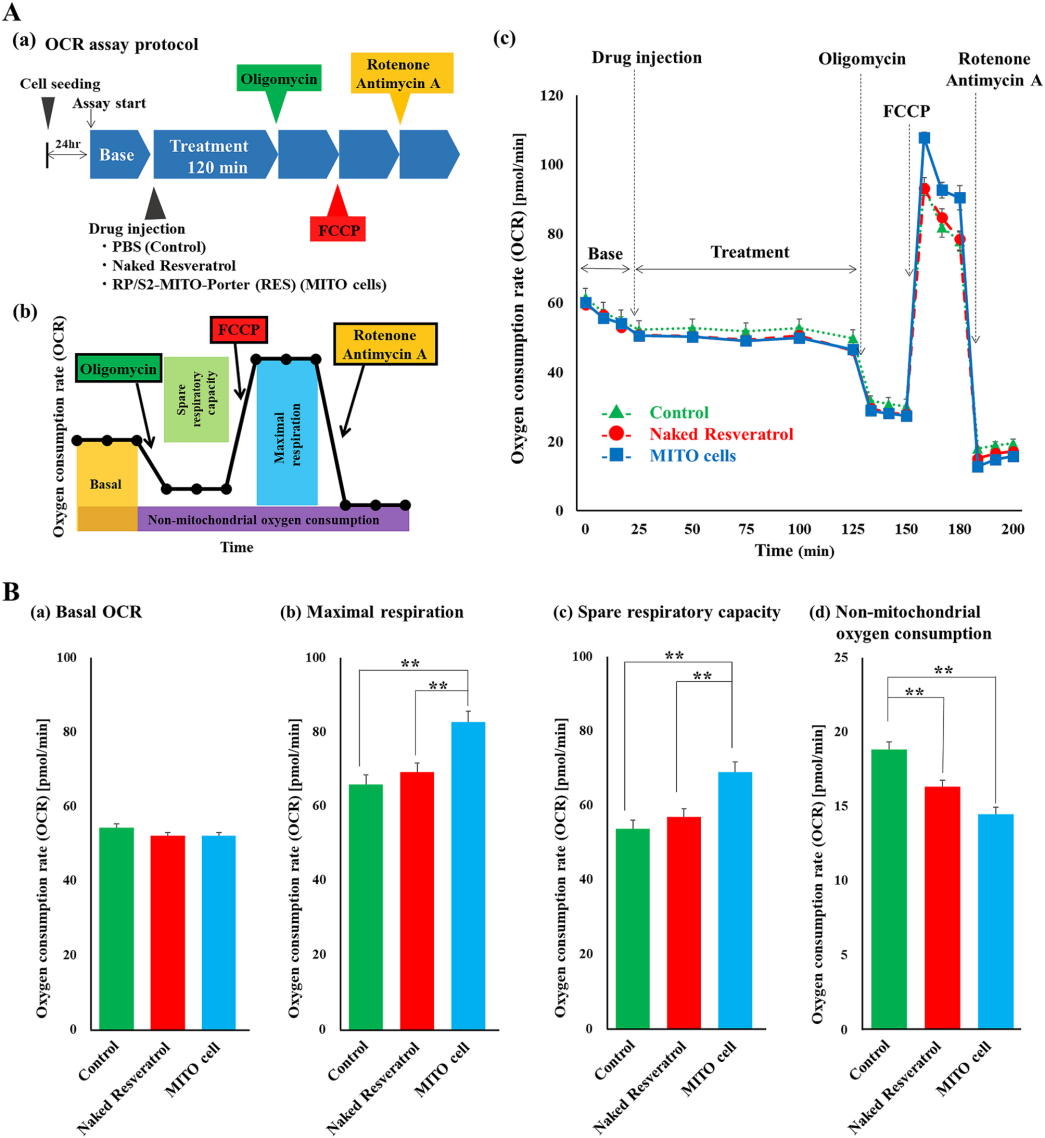
Evaluation of the mitochondrial function of MITO cells by measuring extracellular flux analyzer. The extracellular flux analyzer provides a continuous quantitative assessment of mitochondrial respiration in CPCs by measuring oxygen consumption. The Seahorse FXp was used to measure the mitochondrial oxygen consumption rate (OCR) of CPCs based on the protocol shown in (**A**)(**a**). Basic OCR, ATP-linked OCR, maximum OCR and non-mitochondrial OCR were measured. These parameters were estimated by the sequential addition of oligomycin (an inhibitor of ATP synthesis), carbonyl cyanide p-trifluoromethoxy-phenyl-hydrazone (FCCP) (a mitochondrial inner membrane decarboxylator that maximizes the mitochondrial electron transfer system), and rotenone and antimycin A (the complete inhibition of mitochondrial respiration) and the OCRs were measured (**B**). Significant differences were calculated using one-way ANOVA followed by the SNK test (**p < 0.01) (n = 3).

Mitochondrial function in the untreated CPCs (control group), the CPCs with naked resveratrol and the CPCs with the MITO-Porter (RES) (MITO cells group) were evaluated by measuring mitochondrial oxygen consumption using an extracellular flux analyzer (Fig. 4A).

No change in OCR base line was observed in either group (Fig. 4B(a)). There was a significant increase in Maximal respiration and Spare respiratory capacity in the MITO cells group compared to the control group and the CPCs group with naked resveratrol, indicating an increase in the mitochondrial function of the MITO cells (Fig. 4B(b,c)). The rate of oxygen consumption in the MITO cells group was significantly lower when mitochondrial respiration was stopped than in the control group and the group with naked resveratrol (Fig. 4B(d)). This indicates that oxygen consumption in the MITO cells group was more mitochondria-dependent than in the other two groups.

### Evaluation of cardiac function of ischemic myocardium after cell transplantation

**Figure 5:**
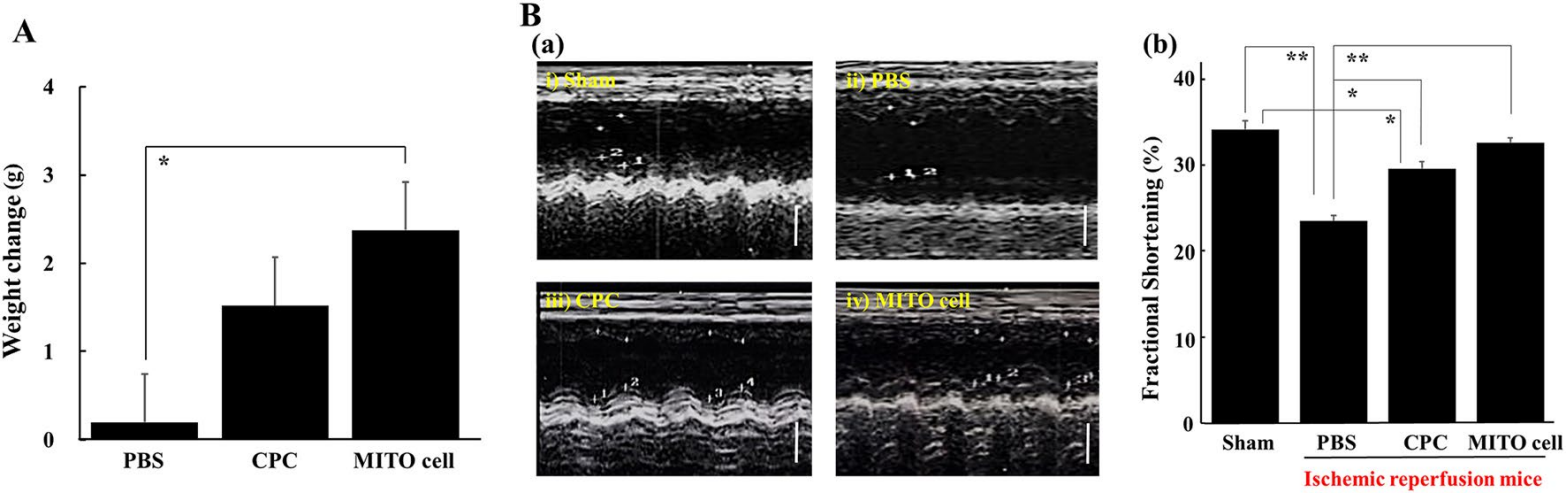
Evaluation of therapeutic effect after cell transplantation. (**A**) Body weight of mice after ischemic perfusion. This figure shows the change in body weight at 30 days after transplantation of each group (PBS treated group (n = 7), CPC group (n = 6), MITO-cell group (n = 6)) in an ischemia–reperfusion mouse model. Data are represented as the mean with S.D. (n = 6–7). *Significant differences were calculated by one-way ANOVA, followed by SNK test (p < 0.05). (**B**) Evaluation of cardiac function of ischemic myocardium. (**a**) Echocardiographic image in 30 days after cell transplantation in ischemia reperfusion model mice. (i) Sham operation mice, (ii) PBS treated, (iii) CPC transplanted and (iv) MITO cell transplanted ischemic reperfusion model mice. Scale bars 2 mm. (**b**) Comparison of fraction shortening among these groups. Fraction shortening (%) was estimated by two-dimension (see “Methods” section for the detail). Sham; Sham operation mice (n = 11), PBS; PBS treated (n = 5), *CPC* CPC transplanted (n = 6) and *MITO cell* MITO cell transplanted (n = 6) ischemic reperfusion model mice group. Data are represented as the mean ± S.D. Significant differences (*p < 0.05, **p < 0.01) were calculated by one-way ANOVA, followed by SNK test.

Ischemia–reperfusion model mice were prepared, and body weight changes were observed at 30 days after MITO cell transplantation, the CPC transplantation or the PBS administration groups (Fig. 5A). The MITO cell transplantation group was + 2.3 g (+ 12.4%), the CPC transplantation group was + 1.5 g (+ 7.4%) and the PBS administration group was + 0.2 g (+ 1%). There was no significant difference between the PBS administration group and the CPC transplantation group, whereas a significant difference was observed between the PBS administration group and the MITO cell transplantation group.

Echocardiography was performed at 30 days after the operation in each of the sham group without ischemic reperfusion, the PBS administration group, the CPC transplantation group, and the MITO cell transplantation group with ischemic reperfusion (Fig. 5B). Fractional shortening rate (FS) was calculated from the left ventricular end-diastolic diameter (LVDd) and left ventricular systolic diameter (LVDs) obtained from the short axis. The values are summarized in Table 2. The rate of shortening of the left ventricle diameter (FS) was 34% in the sham group, 23% in the PBS administration group, 30% in the CPC transplantation group and 33% in the MITO cell transplantation group (Fig. 5B). The value for the CPC transplantation group increased, with a significant difference between that of PBS transplantation group, moreover MITO cell transplantation group improved compared with CPC transplantation group. Comparison of LVDs and LVDd measured in echo between each group was shown in Figure S3.

**Table 2:** Comparison of fraction shortening by two-dimension echocardiography.

	Sham (n = 11)	Ischemic + PBS (n = 5)	Ischemic + CPC (N = 6)	Ischemic + MITO cell (n = 6)
LVDd (mm)	3.16 ± 0.37	3.47 ± 0.59	3.01 ± 0.17	3.32 ± 0.15
LVDs (mm)	2.09 ± 0.32	2.66 ± 0.50	2.12 ± 0.08	2.24 ± 0.17
FS	0.34 ± 0.05	0.23 ± 0.02	0.30 ± 0.17	0.33 ± 0.03
EF	0.62 ± 0.1	0.48 ± 0.03	0.58 ± 0.1	0.61 ± 0.03

Left ventricular cardiac function was evaluated by measuring LVDd, LVDs and FS by echocardiography 30 days after treatment in each group. *LVDd* left ventricular end-diastolic diameter, *LVDs* left ventricular end-systolic diameter, *FS* fractional shortening, *EF *ejection fraction.

### Evaluation of myocardial tissue fibrosis after CPC transplantation and MITO cell transplantation in ischemia–reperfusion model mice

**Figure 6:**
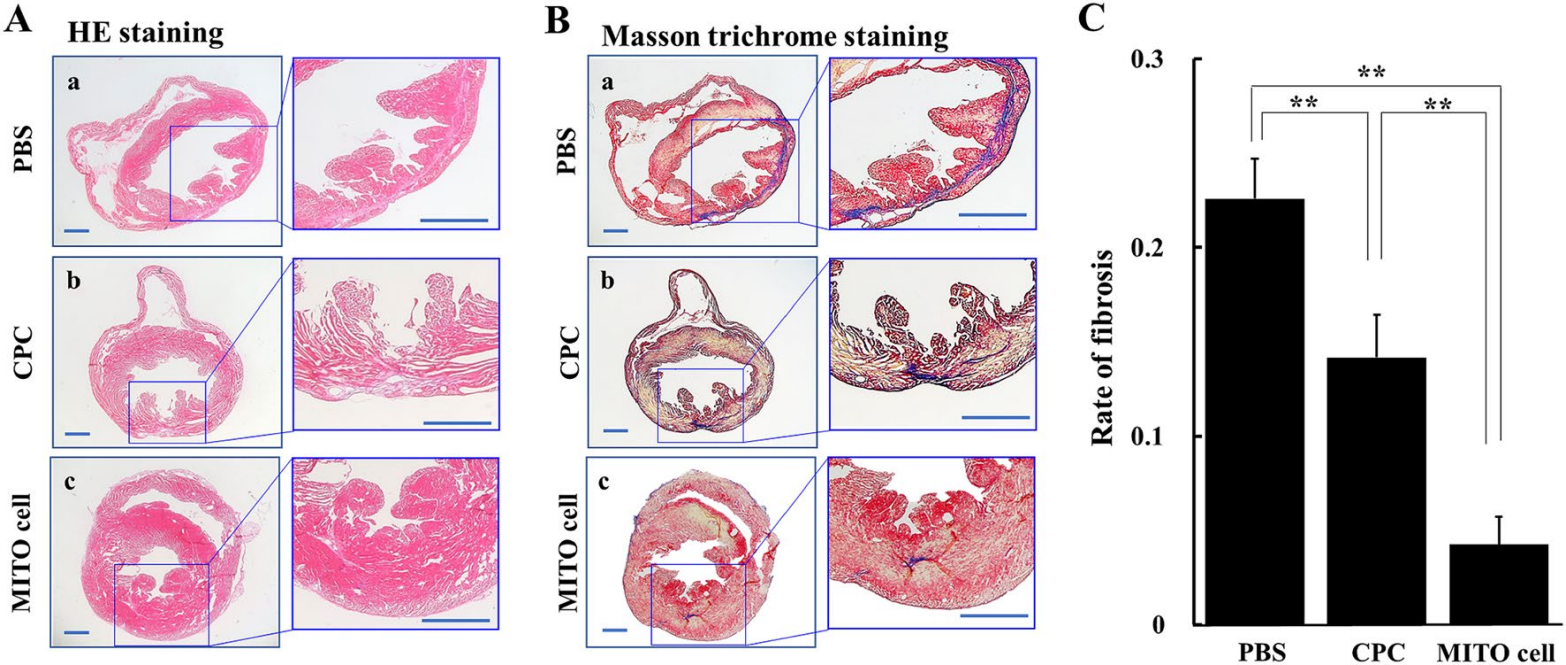
HE and Masson trichrome straining in ischemic myocardium after cell transplantation. HE staining (**A**) and Masson trichrome staining (**B**) in sections of ischemic myocardium 30 days after treatment in each group (PBS group (**a**), CPC group (**b**), MITO-cell group (**c**)). Left panels are magnified images of interest of region. Scale bar is 200 μm. (**C**) The photographic images shown in (**B**) were analyzed to determine the rate of fibrosis using Image-pro Plus 7.0. PBS, PBS treated; CPC, CPC transplanted; MITO cell, MITO cell transplanted. Data are represented as the mean ± S.D (n = 5). Significant differences (**p < 0.01) were calculated by one-way ANOVA, followed by the SNK test.

Ischemia–reperfusion model mice were prepared, and the hearts were excised at 30 days after the PBS administration group, the CPC transplantation group, and the MITO cell transplantation group. Then histopathological tissue sections were prepared for Hematoxylin–Eosin (HE) (Fig. 6A) and Masson trichrome staining (Fig. 6B).

In the PBS administration group, extensive fibrosis was observed in the anterior wall region of the left ventricle, whereas in the CPC transplantation group, the degree of fibrosis was suppressed compared to that for the PBS administration group. Fibrosis was further suppressed in the MITO cell transplantation group compared to the CPC transplantation group, indicating that the extent of fibrosis was most suppressed in the MITO cell group. The photographic images shown in Fig. 6B were analyzed to determine the rate of fibrosis (Fig. 6C). The results of the image analysis indicated that there were significant differences between the MITO cell treatment group and the other groups.

### Evaluation of oxidative stress and mitochondrial membrane potential after cell transplantation in ischemia reperfusion model mouse

**Figure 7:**
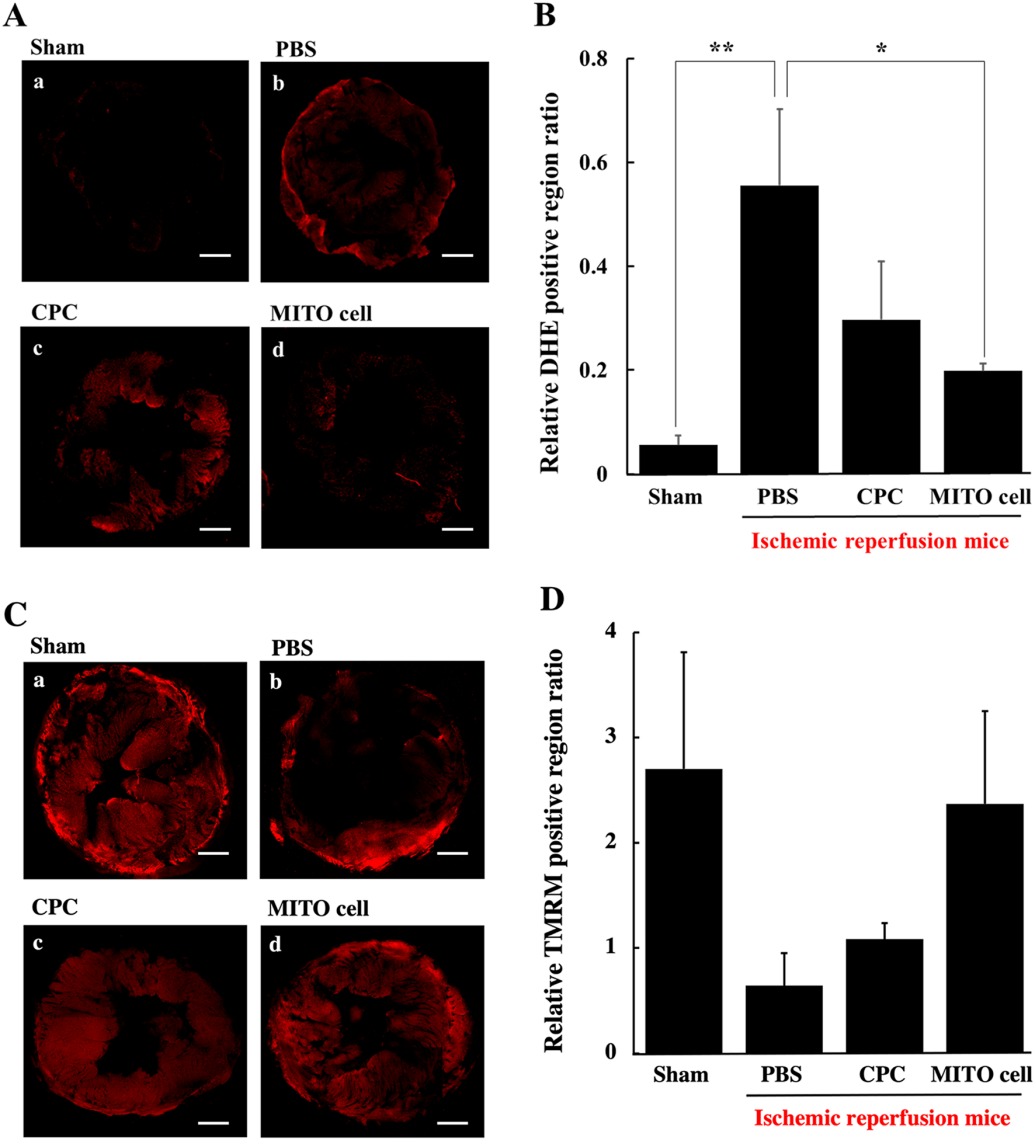
Detection of oxidative stress of ischemic myocardium after cell transplantation. (**A**) CLSM observation of oxidative stress of ischemic myocardium 3 days after treatment in each group using DHE (a red dye for reactive oxygen spices). (**a**) Sham group, (**b**) PBS group, (**c**) CPC transplantation group and (**d**) MITO-cell transplantation group. Scale bars, 200 μm. (**B**) Quantification of relative DHE positive region ratio was estimated using CLSM images. Data are represented as the mean ± S.D (sham group (n = 6), PBS group (n = 4), CPC transplantation group (n = 4) and MITO-cell transplantation group (n = 4)). Significant differences (*p < 0.05, **p < 0.01) were calculated by one-way ANOVA, followed by SNK test. Scale bar is 200 μm. Detection of mitochondrial membrane potential of ischemic myocardium after cell transplantation. (**C**) CLSM observation of mitochondrial membrane potentials of ischemic myocardium 3 days after treatment in each group using TMRM (a red dye for mitochondrial membrane potential). (**a**) Sham group, (**b**) PBS group, (**c**) CPC transplantation group and (**d**) MITO-cell transplantation group. Scale bars 200 μm. (**D**) Quantification of relative TMRM positive region ratio was estimated using CLSM images. Data are represented as the mean ± S.D (sham group (n = 6), PBS group (n = 5), CPC transplantation group (n = 5) and MITO-cell transplantation group (n = 5)). Significant differences (*p < 0.05, **p < 0.01) were calculated by one-way ANOVA, followed by SNK test.

Ischemia–reperfusion model mice were prepared for the PBS administration group, the CPC transplantation group and the MITO cell transplantation group. A control group without ischemia (Sham group) was also prepared. At 3 days after the transplantation, the hearts of the mice were excised, and the levels of ROS in the myocardial tissue was visualized using DHE to quantify the relative DHE positive region ratio (Fig. 7A,B). Compared to the Sham group, the PBS administration group showed a higher increase in the relative DHE positive region ratio. Whereas ROS levels were suppressed in the case of the CPC and MITO cell transplantation groups. Moreover, the value was suppressed in the MITO cell transplantation group and this difference was significant compared to the PBS administration group.

Using the same model, mitochondrial membrane potentials of myocardial tissue were measured by TMRM, a red fluorescent dye to detect mitochondrial membrane potential, followed by quantification of the relative TMRM positive region ratio (Fig. 7C,D). Compared with the sham group, the PBS administration group showed a dramatic reduction in the mitochondrial membrane potential levels in the infarcted site, while the MITO cell group was maintained the mitochondrial membrane potential. These results indicate that the MITO cell transplantation group showed the greatest suppression of ROS generation and retention of mitochondrial membrane potential levels.

## Discussion

There have been numerous reports of cardiomyocyte stem cell transplantation for the treatment of heart failure using untreated CPCs or drug treated CPCs without DDS, and studies in mouse models have shown therapeutic effects^20,21^. Many clinical studies have been conducted regarding cell transplantation in humans using CPC and mesenchymal stem cells (MSC). Most of them have few adverse events and are somewhat effective. However, due to the low fixation rate of transplanted cells and the problems associated with the transplantation method^22^, cell transplantation therapy has been not yet reached the clinical stage. Based on this background, we concluded that the function of the cells themselves need to be enhanced if this cell transplantation therapy is to reach the stage of clinical application. We previously could prepare mitochondria activated myocardial stem cells (MITO cell) by the mitochondrial delivery of resveratrol using a MITO-Porter (RES)^19^. In addition, we demonstrated an improvement in the transplantation of MITO cells in a mouse model of doxorubicin cardiomyopathy compared to conventional CPC transplantation methods, thus demonstrating the preventive effect of MITO cell transplantation^19^. In an experimental protocol to validate the preventive effect, the drug is administered before the onset of the disease condition. While, a validation of the therapeutic effect uses a protocol in which the drug is administered after the onset of the disease. In general, experiments to obtain a therapeutic effect are more difficult to achieve than experiments to obtain a preventive effect. The purpose of this study is the validation of the therapeutic effect of MITO cell transplantation, which is different from the examination of preventive effects of MITO cell transplantation, previously reported^19^.

In this study, we attempted to improve the intracellular trafficking of the MITO-Porter (RES) to further enhance its therapeutic effects. It has been reported that, when the surfaces of carriers are modified with the S2 peptide, that this increases their intracellular uptake and mitochondrial migration^17^. In addition, in the case of an R8-modified MITO-Porter, it has been reported that the process of cellular uptake and mitochondrial targeting is enhanced by modifying the particle with an RP aptamer^23^. In the present study, the surface of the S2-MITO-Porter (RES) was modified with an RP aptamer, and cellular uptake was then evaluated. The results showed that the RP/S2-MITO-Porter (RES) showed the highest increase in cellular uptake (Fig. 3). This suggests that modifying the surface of the particle with the RP aptamer enhanced the cellular uptake of the MITO-Porter in the presence of the S2 peptide as well as the R8 peptide. Although we did not evaluate the cellular uptake of resveratrol, in a previous study, using transmission electron microscopy analyses, we confirmed the accumulation of gold colloids in the mitochondria, when gold colloids encapsulated the MITO-Porter was added to the cells^10^. We also observed that intracellular mitochondrial function was improved due to the enhanced cellular uptake capacity caused by the RP/S2-MITO-Porter (RES) (Fig. 4). Thus, we considered that this resulted from the increased cellular uptake of the resveratrol drug itself resulting in an enhanced mitochondrial accumulation of resveratrol.

Similar to our previous report^24^, S2 peptide modification allowed for the intracellular uptake of the MITO-Porter (RES) in the presence of serum. In the case of the R8-MITO-Porter, serum-free medium was used for the cellular uptake, because the carriers did not maintain their stability and aggregated in serum-containing medium, resulting in a significant decrease in cell uptake ability. Culturing cells in a serum-free medium induces cell starvation and might cause cytotoxicity, which is one of the strategies for improving cell transplantation process. The S2-MITO-Porter (RES) could be used in a cell culture medium that contained serum, thus allowing transplanted cells to be cultured in a natural environment. This is an important point for future clinical applications, and the achievement of a stable uptake in the presence of serum has made it possible to conduct this type of research in a more clinically relevant manner.

In clinical practice, if a patient has an acute myocardial infarction, a coronary artery catheterization is performed immediately after the patient arrives at the hospital. The infarct site is typically identified, followed by the reperfusion of coronary blood flow. The possible clinical timing of the cell therapy is intracardiac administration using a catheter after the release of myocardial ischemia. In clinical studies, intracoronary cell-based transplantation is often the treatment of choice as an add-on to reperfusion therapy. Cell transplantation has been successfully used in clinical trials to improve the conditions associated with reperfusion and to protect the myocardium^25^.

In this study, we prepared coronary artery ischemia–reperfusion model mice, and examined the therapeutic effects of cell transplantation on the treatment of heart failure. In the operation, CPCs and MITO cells were directly injected into the myocardium during the acute phase of ischemia–reperfusion. At 30 days after the cell transplantation, the MITO cell group had the best weight gain and a good left ventricular function on echocardiography (Fig. 5). In terms of weight change (Fig. 5A), there was no significant difference between the CPC group and the control group (PBS group), but there was more weight gain in the MITO cell group than in the CPC group. Moreover, there was a significant difference between the MITO cell and PBS groups (p < 0.01), although there was no significant difference between the CPC and PBS groups. Echocardiography (Fig. 5B) findings showed a significant difference between the MITO cell and PBS groups (p < 0.01). In the comparison between CPC and PBS groups, there is also a significant difference (p < 0.05), but the value of FS is higher in the MITO cell group than in the CPC group. Furthermore, there was no significant difference between the MITO cell and the Sham groups, but there was a significant difference between the CPC and the Sham groups (p < 0.05). The fibrosis rate of the tissues also showed a significant suppression of fibrosis in pathological tissues in the MITO cells group compared to the CPC group (p < 0.01) (Fig. 6), suggesting that the damage to the myocardium caused by ischemia was minimal. These results indicate a positive therapeutic benefit of MITO cells compared to CPC transplants.

Mitochondrial function in myocardial tissue was evaluated at 3 days after cell transplantation, the MITO cells suppressed ROS generation, and an evaluation of the membrane potential showed that the effect was the same as that for the ROS evaluation (Fig. 7C,D). The MITO cell group suppressed the generation of ROS, not only at the site where the cell was transplanted but also throughout the tissue. It is unlikely that the transplanted CPCs themselves proliferated and differentiated into myocardium cells in this short period of time, and other mechanisms of action cannot be excluded. The mechanism responsible for the therapeutic effect that was conferred on CPC-derived MITO cells is thought to be that MITO cells originally possessed the same therapeutic effect as CPCs due to the paracrine effect^19,26,27^. In the case of stem cell transplantation, the paracrine factor would be expected to prevent cardiomyocyte death caused by oxidative stress. In stem cell transplantation, it has been reported that cell adhesion leads to a paracrine effect, followed by cell migration and neovascularization^26^.

It is possible that some factor induced by the action of CPCs caused an antioxidant effect that suppressed the generation of ROS throughout the tissue and maintained the mitochondrial membrane potential, and further enhanced the action in MITO cells^28^. Another possibility is mitochondrial transfer, in which stem cells migrate from their own mitochondria to other injured tissue cells thus optimizing the intracellular environment^29^.

We found that mitochondrial function increased more in MITO cells than in CPCs, as evidenced by measurements made using an extracellular flux analyzer (Fig. 4). These findings suggest that the enhancement in mitochondrial function of MITO cells would contribute to an increase in viability and a more robust paracrine effect and mitochondrial transfer. By inducing such actions, it is likely that the effect of this action was not localized but, rather, was more widespread in terms of the improvement of the tissue. Although antioxidant effects and tissue mitochondrial membrane potential retention were clearly observed, the detailed mechanisms responsible for this were not fully elucidated in this study. Additional analysis will be needed to confirm that MITO cells show an antioxidant effect by measuring inflammatory cytokines in transplanted individuals, the degree of DNA damage in the transplanted tissues, and mRNA expression of ROS markers. Furthermore, by observing MITO cell-derived mitochondrial dynamics in tissues, we believe that mitochondrial transfer could be further analyzed. The elucidation of the mechanisms that are involved in this process will allow us to develop additional therapies in addition to conventional cell transplantation therapy. Activation of the transplanted cells would be expected to increase the cell transplantation efficiency. This is likely to help improve the weaknesses that appear to be inherent in conventional cell transplantation therapy for clinical applications. As a future objective, we plan to conduct experiments with CPCs derived from humans instead of that from mouse for clinical applications.

## Methods

### Materials

Resveratrol was purchased from Wako Pure Chemical Industries, Ltd. (Osaka, Japan). 1,2-Dioleoyl-sn-glycero-3-phosphatidyl ethanolamine (DOPE) and DOPE-N-(7-nitro-2-1,3-benzoxadiazole-4-yl) (NBD-DOPE) were purchased from Avanti Polar Lipids (Alabaster, Alaska). Sphingomyelin (SM) was purchased from Sigma (St. Louis, Missouri). Stearylated S2 peptide (STR-S2, stearylated (Dimethyl-Tyr)-(D-Arg)-(Phe)-(Lys)-(Dimethyl-Tyr)-(D-Arg)-(Phe)-(Lys)-NH2) was obtained from the Toray Research Center, Inc. (Tokyo, Japan). Cholesteryl RP aptamer (Chol-RP, cholesteryl 5′-CUCCCUGAGCUUCAGG-3′) was purchased from Greiner bio-one (Tokyo, Japan). Dihydroethidium (DHE), Dulbecco's modified Eagle's medium F-12 (DMEM F-12), fetal bovine serum (FBS), MitoTracker^®^ Deep Red FM and Tetramethylrhodamine, methyl ester (TMRM) were purchased from Thermo Fisher Scientific Life Sciences (Waltham, Massachusetts). Hoechst 33342 was purchased Dojindo molecular Technologies, Inc. (Kumamoto, Japan). All other chemicals used were commercially available, reagent grade products.

### Experimental animals

C57BL/6J male mice (8–10 weeks old) were purchased from Sankyo labo Service (Tokyo, Japan) and housed at a 12 h light–dark cycle with free access to water and standard mouse food in a specifically pathogen-free room. In a previous report on the validation of the preventive effect of MITO Cell using DOX-induced cardiomyopathy mice, the number of samples was set at 6 and statistical analysis could be performed^19^. Therefore, at the beginning of the animal experiment in this study, we estimated that the number of samples would be 6 and proceeded with the experiment. The number of cases reaching the final stage, excluding those that were lost due to surgical or anesthetic errors was 4–6. From an animal welfare point of view, it was decided to evaluate the small number of cases for which a statistical analysis could be performed. Euthanasia was performed by cervical dislocation while isoflurane inhalation anesthesia was used in cases of sufficient activity in all cases. All experimental procedures were performed according to the animal welfare regulations of Hokkaido University. All animal protocols were approved by the institutional animal care and research advisory committee of the Faculty of Pharmaceutical Sciences, Hokkaido University, Sapporo, Japan (date: 28 March 2016, registration no. 16-0015).

### Construction of RP/S2-MITO-Porter (RES)

We prepared the RP/S2-MITO-Porter (RES) by the lipid film hydration method refer to our previously report 19. A suspension containing 122.5 μL of chloroform containing 137.5 nmol lipids [DOPE/SM 9:2 (molar ratio)], including 50 nmol resveratrol was prepared in a glass tube. After evaporation of the organic solvent, the lipids were hydrated in HEPES buffer (pH 7.4). The resulting suspension was then subjected to sonication using a bath-type sonicator (85 W; Aiwa Company, Tokyo, Japan) to prepare the RES contained plane liposome (DOPE/SM-LP (RES)) without cellular uptake activity. The DOPE/SM-LP was mixed with an STR-S2 (10 mol% of total lipid) and RP aptamer (2 mol% of total lipid) to prepare the RP/S2-MITO-Porter (RES). The S2-MITO-Porter (RES) without a RP aptamer was also prepared as a control carrier. The diameters and ζ-potentials of these carriers were measured using a Zetasizer Nano ZS instrument (Malvern Instruments, Worcestershire, UK) (Table 1). Their intracellular trafficking was determined using carriers that were labelled with NBD-DOPE (1 mol% of total lipids) (Fig. 3).

### Isolation and culturing of CPCs and the preparation of MITO cells

As shown in Figure S1 (Supporting Information) 19, mice were anesthetized by inhalation anesthesia, euthanasia was performed by cervical dislocation, and the ventricular part was removed rapidly (Step 1). The collected myocardium was rinsed with buffer and then treated with collagenase to disperse the cells (Step 2). The cells were separated using density gradient centrifugation, cell layers were collected and seeded into collagen-coated dishes, followed by incubation at 37 °C, under an atmosphere of 5% CO_2_ (Step 3). After 2–3 passages, magnetic cell sorting (MACS) was performed using the Sca-1 antibody to collect Sca-1 antibody-positive cells (Step 4). The obtained cells were positive for Sca-1, CD29, and CD106 as mesenchymal stem cell markers and negative for CD117 and CD45 (Fig. S1), thus confirming that the cells were, in fact, stem cells. CPCs were cultured 3–4 times in culture medium supplemented with growth factor (FGF). In addition to harvesting stem cells using the same technique as in previous reports^19^, we confirmed that the cells were, in fact, stem cells by reconfirming the positivity of the stem cells isolated from these cardiomyocytes for mesenchymal stem cell markers before using them as transplantation cells. MITO cells were prepared to activate the CPCs by mitochondrial delivery of resveratrol using a MITO-Porter system as shown in Figure S2 (Supporting Information). Detailed information regarding these experimental processes can be found in the Supporting Information.

### Intracellular trafficking analysis of the carriers in CPCs

After adding the RP/S2-MITO-Porter (RES), S2-MITO- Porter (RES) or DOPE/SM-LP (RES) labeled with NBD-lipids to the CPCs, they were then incubated for an hour, followed by measuring cellular uptake by flow cytometry (Gallios; BEKMAN COULTER (Tokyo, Japan)) and Kallza software (BEKMAN COULTER), as previously reported^30^. The cellular uptake is expressed as the mean fluorescence intensity (MFI). Moreover, the intracellular localization of these carriers was observed using confocal laser scanning microscopy (CLSM) (FV-10 Olympus, Tokyo, Japan). The cells were excited with a 473 nm light and a 635 nm light from an LD laser. Images were obtained using an FV10 equipped with a water-immersion objective lens (UPlanSApo 60×/NA. 1.2) and a dichroic mirror (DM405/473/559/635). The two fluorescence detection channels (Ch) were set to the following filters: Ch1:420/40 (green) for NBD, Ch2: 660/50 (red) for Mitotracker Deep Red.

In this observation, when green labeled carriers (green color) were co-localized with red stained mitochondria, yellow signals were observed in the merged image. Detail information for the experiment regarding the cellular uptake can be found in the Supporting Information.

### Evaluation of the mitochondrial function of MITO cells by measuring extracellular flux analyzer

The extracellular flux analyzer provides a continuous quantitative assessment of mitochondrial respiration in CPCs by measuring oxygen consumption. The Seahorse FXp (Agilent Technologies, Santa Clara, CA, USA) was used to measure the mitochondrial oxygen consumption rate (OCR) of CPCs based on the protocol shown in Fig. 4A(a). Basic OCR, ATP-linked OCR, maximum OCR and non-mitochondrial OCR were measured. These parameters were estimated by the sequential addition of oligomycin (an inhibitor of ATP synthesis), carbonyl cyanide p-trifluoromethoxy-phenyl-hydrazone (FCCP) (a mitochondrial inner membrane decarboxylator that maximizes the mitochondrial electron transfer system), and rotenone and antimycin A (the complete inhibition of mitochondrial respiration) and the OCRs were measured (Fig. 4B). At 24 h before the start of the experiment, 20,000 CPCs per well were seeded into 8-well plates and incubated at 37 °C under an atmosphere of 5% CO_2_. Prior to the analysis, the cells were incubated in analytical medium at 37 °C and CO_2_-free for 1 h. The cells were divided into three groups: a PBS treated group (control group), a naked resveratrol group and a MITO cells group (the group to which MITO cells were made by adding RP/S2-MITO-Porter (RES)). The samples containing resveratrol (final conc., 10 μM) were added to the cells after measuring the baseline OCR. After incubation for 120 min, oligomycin (final concentration of 1 μM), FCCP (final concentration of 1.5 μM), rotenone (final concentration of 0.5 μM) and antimycin A (final concentration of 0.5 μM) were continuously added to the cells (Fig. 4A(a,b). The obtained data (Fig. 4A(c)) were used to determine the OCR [pmol/min] as the mitochondrial respiratory activity. Figure 4B shows data regarding Basal OCR, Maximal respiration, Spare respiratory capacity and non-mitochondrial oxygen consumption.

### Transplantation of MITO cells to the heart in myocardial ischemia/reperfusion injury mouse and validation of the therapeutic effect

C57BL/6 mice were anesthetized (inhalation anesthetic apparatus: NARCOBIT-E type II NATSUME SEISAKUSHO (Tokyo, Japan)) and ventilated (KN-58 SLA Ventilator: NATSUME SEISAKUSHO) with 2% isoflurane after intubation. During surgery, a hot pad (NATSUME SEISAKUSHO) was used to prevent a drop in body temperature, and an electrocardiographic monitor (Softron (Tokyo, Japan)) was used as a vital monitor. The mouse was fixed in a supine position and a thoracotomy was then performed (Fig. 1). The left anterior descending coronary (LAD) was exposed, and a 8-0 needle-nylon (Kono Seisakusho Co., Ltd. (Tokyo, Japan)) thread was used to attach a thread to the myocardium below the coronary artery. The thread was then ligated so as to prepare a sandwich PE-10, and ischemia of LAD was then performed. Particular care was taken during ligation. We rigorously confirmed that the anatomical coronary arteries were ligated in the same place, by visually checking for color changes in the myocardium, and for changes in ischemia by electrocardiograms. These methods were used to check for the presence of and the extent of myocardial ischemia each time. Ischemia was performed for 60 min, the ligation stopped and reperfusion initiated (Fig. 1). In order to avoid bias in model generation, the treatment-to-transplant cycles (PBS group, CPC group, and MITO cell group) were pre-determined before the operations were performed.

After confirming the improvement of the general condition of the mouse and the ECG change, only PBS, 1 × 10^6^ MITO cells, or 1 × 10^6^ CPCs suspended in 30 μL PBS were injected at the three sites within the left ventricle. Changes in body weight changes 30 days before and after transplantation and wall motion of the left ventricle 30 days after surgery were evaluated by echocardiography. For the echocardiographic procedure, mice were sedated under inhalation anesthesia and short-axis images of the left ventricle were collected. Fractional shortening rate (FS) was calculated from the left ventricular end-diastolic diameter (LVDd) and left ventricular systolic diameter (LVDs) obtained from the short axis. The values are summarized in Table 2. There were cases of death before follow-up due to intraoperative surgical errors or anesthesia errors. Since these issues were observed before follow-up, they were excluded completely from the study. On the other hand, mice that had completed the ischemia treatment and cell transplantation did not die during follow-up. In order to avoid subjectivity in the evaluation, the order of evaluation was randomized, and the final analysis was done by matching the numbers.

The hearts were sectioned and subjected to HE staining and Masson trichrome staining to evaluate the extent of cardiac fibrosis. The excised hearts were fixed with paraformaldehyde. After dehydration, they were frozen and sectioned and 10 μm thick samples prepared. The resulting sections were subjected to hematoxylin–eosin staining. Masson trichrome staining was performed on the same sections to evaluate the degree of fibrosis. The assessment of fibrosis was performed at the level of the papillary muscle of the left ventricle so that the cutout position was constant^31^. In Masson trichrome staining, iron hematoxylin stains the nucleus black, acid fuchsin stains the cytoplasm red, and aniline blue stains the cytoplasm red. Collagen fibers are stained blue with aniline blue. This indicates that the blue-colored area is where the fibrosis occurs. Masson trichrome stained tissue sections were analyzed to determine the rate of fibrosis. The photographic images were subjected to image analysis using Image-pro Plus 7.0. The fibrosis rates were calculated by dividing the area of fibrosis in the ventricle (stained blue) by the area of the entire myocardial tissue.

### Evaluation of ROS and mitochondrial membrane potential after cell transplantation in ischemia–reperfusion model mice using CLSM

After the MITO cell transplantation, ROS production was evaluated by CLSM. Ischemia–reperfusion model mice were prepared and divided into a control group without ischemia, a PBS group, a CPC transplantation group, and a MITO cell transplantation group. Three days after creating the models, mouse hearts were extracted. The extracted hearts were sectioned at the level of the short-axis papillary muscle, and stained with DHE (final concentration 5 μM) to detect ROS and Hoechst 33342 (final concentration 10 μM) to stain the nucleus in PBS at 4 °C for 30 min. After incubation, the tissues were washed with PBS, and then observed by CLSM (Nikon A1; Nikon Company Ltd., Tokyo, Japan). The tissues were excited by a 405 nm light from a diode laser, 561 nm light from an Ar laser. Images were obtained using a Nikon A1 equipped with an objective lens (Plan Apo 10×/0.45) and a 1st dichroic mirror (405/488/561/640). The two fluorescence detection channels (Ch) were set to the following filters: BP (450/50) (blue color) for Hoechst 33342, and Ch2: BP 525/75 (red color) for DHE.

Mitochondrial membrane potential was evaluated using TMRM following the same method as described above. Each model was stained with TMRM (final concentration 250 nM) and Hoechst 33342 (final concentration 10 μM) in PBS at 4 °C for 30 min. Images were obtained using a Nikon A1 equipped. The two-fluorescence detection Chs were set to the following filters: Ch1: BP (450/50) (blue color) for Hoechst 33342, Ch2: BP (525/50) (red color) for TMRM.

The photographed images were subjected to image analysis using Image-pro Plus 7.0. DHE and TMRM fluorescence (red color) per area was determined by dividing the red integrated density by the blue integrated density. The integral optical density of DHE and TMRM (red) was calculated, and the integral optical density of Hoechst 33342 (blue) was then calculated. The integrated optical density of blue of Hoechst 33342 was divided by the integrated optical density of red of DHE and TMRM. The intensity of DHE and TMRM per cell component of one section was calculated. Intensity of the ROS = (integral optical density of DHE (red))/(integral optical density of Hoechst 33,342 (blue)). Intensity of the membrane potential = (integral optical density of TMRM (red))/(integral optical density of Hoechst 33,342 (blue)).

### Statistical analysis

Data are expressed as the average value with the standard deviation (S.D.) for the indicated number of experiments. For multiple comparisons, one-way ANOVA was performed, followed by the Student–Newman–Keuls test. Levels of p < 0.05 were considered to be significant.

### Ethical approval

This work is licensed under a Creative Commons Attribution 4.0 International License. The images, movies or other third-party material in this article are included in the article’s Creative Commons license, unless indicated otherwise in the credit line; if the material is not included under the Creative Commons license, users will need to obtain permission from the license holder to reproduce the material. To view a copy of this license, visit http://creativecommons.org/licenses/by/4.0/.

### Animal experiments

The use of the mice was approved by the Ethics of Pharmaceutical Science Animal Committee of Hokkaido University (approval number: 16-0015). All experiments were performed in accordance with National University Corporation Hokkaido University Regulations on Animal Experimentation and with the ARRIVE guidelines.

## Supplementary Information

Supplementary Information 1.

Supplementary Information 2.
