# Supplementary material for: Transplantation of MITO cells, mitochondria activated cardiac progenitor cells, to the ischemic myocardium of mouse enhances the therapeutic effect
Source: Sci Rep. 2022 Mar 22;12:4344. doi: 10.1038/s41598-022-08583-5 (PMC8941106; doi:10.1038/s41598-022-08583-5)
Supplement: Supplementary file 1 — Supplementary Information 1. [file 41598_2022_8583_MOESM1_ESM.pdf]

supplementary **SUPPLEMENTARY INFORMATION**

Supplementary Methods

1. Isolation and culturing of CPCs.

Stem cell antigen (Sca)-1 positive CPCs were isolated from the hearts of C57B/6 male mice and cultured, as previously reported [1]. Hearts from 8 to 10-week-old C57B/6 male mice were minced and treated twice with a 0.2% solution of type II collagenase and (Worthington Biochemical Corp., Lakewood, NJ, USA) for 30 min at 37°C. Isolated cells were size-fractionated with a 30%–70% Percoll gradient to produce single-cell suspensions that were devoid of debris and fibrotic tissues. Purified cells were cultured in DMEM F-12 supplemented with 10% FBS, penicillin/streptomycin, and a 40 ng/mL solution of mouse recombinant basic fibroblast growth factor (bFGF) (R&D Systems, Minneapolis, Minnesota) at 37°C and 5% CO_2. Expanded cells were incubated with Sca-1 antibody conjugated magnetic beads (Miltenyi Biotec, Bergisch Gladbach, Germany) to obtain a pure population of Sca-1 positive CPCs by magnetic activated cell sorting [2]. The quality of Sca-1 positive CPCs was evaluated using a procedure described in a previous report [3].

2. Preparation of MITO cell

MITO cells were prepared to activate the CPCs by the mitochondrial delivery of resveratrol using a MITO-Porter system, as shown in Figure S2 based on the method of previous report [1]. Breifly, purified Sca-1 positive CPCs (1×10^6 cells/well) were cultured in 10 mm collagen-coated dish (Iwaki; ASAHI GLASS co., Ltd., Tokyo) with DMEM F-12 supplemented with 10% FBS, penicillin/streptomycin, and 40 ng/mL mouse recombinant bFGF under an atmosphere of 5% CO_2/air at 37 °C for 24 hr. The cells were then additionally incubated in DMEM F-12 with added serum in the presence of the MITO-Porter (RES) for 2 hr. The final concentration of resveratrol was 10 μM in the case of *in vitro* and *in vitro* experiments. Before using MITO cells for *in vitro* and *in vivo* experiments, they were purified to remove the residual MITO-Porter (RES) that was attached on the surface of the CPCs. Briefly, after incubating the CPCs with the MITO-Porter (RES), the cells were detached from the culture plate *via* a trypsin treatment and the cell suspension was then centrifuged at 300 *g* for 3 min at 4°C. After removing the supernatant, the pellet was resuspended in PBS. The resulting suspension was used as the MITO cell suspension and was stored on ice before transplantation.

3. Cellular uptake analysis.

Cellular uptake of the RP/S2-MITO-Porter (RES), the S2-MITO-Porter (RES) or the DOPE/SM-LP (RES) by CPCs were evaluatd by flow cytometry, as previously reported [4]. CPCs (1×10^5 cells/well) were seeded on a six-well collagen-coated plate (Iwaki) with DMEM F-12, containing 10% FBS under an atmosphere of 5% CO_2/air at 37°C for 24 hr. The NBD-labelled carriers (final concentration of total lipid, 13.75 μM) were added to the CPCs. The cells were then incubated in serum-free medium for 1 h, and the medium was then removed. After washing the cells, they were analyzed by flow cytometry (Gallios; BEKMAN COULTER (Tokyo, Japan)) and Kallza software (BEKMAN COULTER),. The cells were excited with a 488 nm light, and the fluorescence detection channel was set to a 530 nm FL1 filter. Cellular uptake is expressed as the mean fluorescence intensity (MFI).

Supplementary Figure

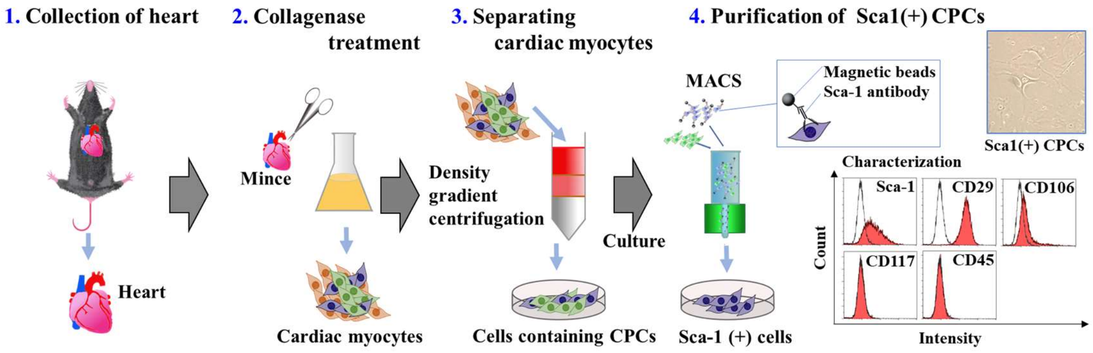

Figure S1. Preparation of cardiac progenitor cells (CPCs) from mice heart tissue.

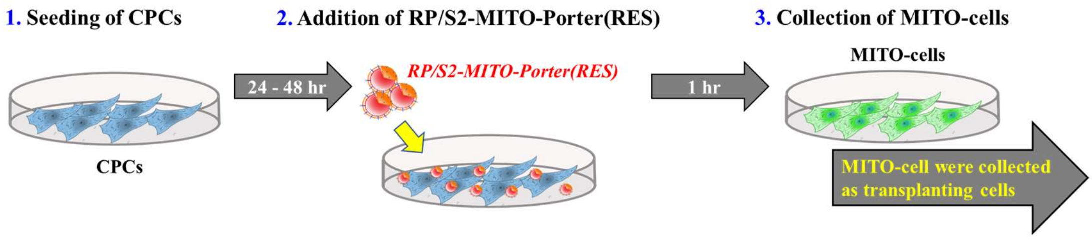

Figure S2. Procedure used to prepare mitochondria activated CPCs (MITO cells) by treatment with the RP/S2-MITO-Porter (RES).

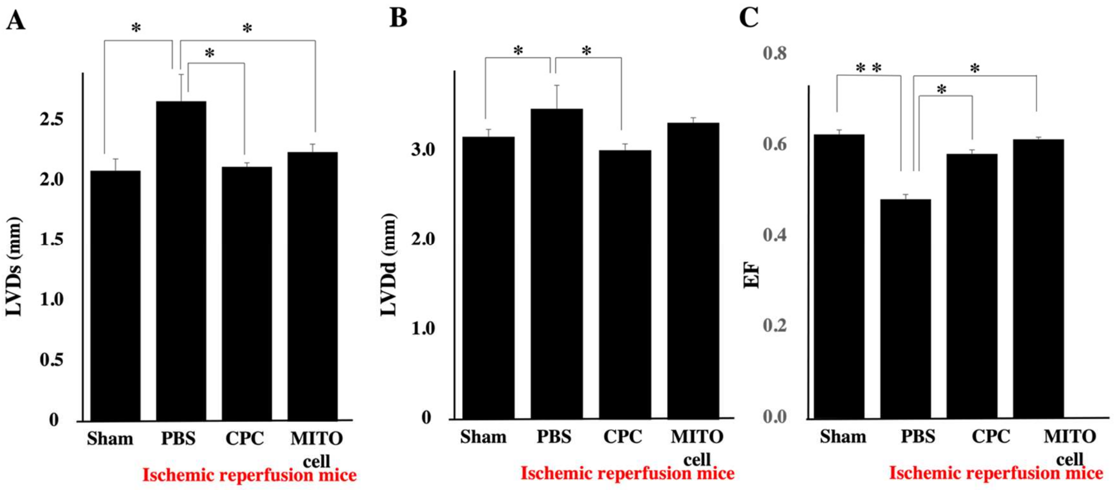

Figure S3. Comparison of LVDs and LVDd measured in echo between each group. Data are expressed as the mean ± S.D. (n = 5-11). Significant differences (*$p < 0.05$, **$p < 0.01$) were calculated by SNK test followed by one-way ANOVA. LVDd: left ventricular end-diastolic diameter, LVDs: left ventricular end-systolic diameter, EF: ejection fraction.

References

1. Abe J, Yamada Y, Takeda A and Harashima H. Cardiac progenitor cells activated by mitochondrial delivery of resveratrol enhance the survival of a doxorubicin-induced cardiomyopathy mouse model via the mitochondrial activation of a damaged myocardium. *J Control Release*. 2018;269:177-188.
2. Smirnov A, Comte C, Mager-Heckel AM, Addis V, Krasheninnikov IA, Martin RP, Entelis N and Tarassov I. Mitochondrial enzyme rhodanese is essential for 5 S ribosomal RNA import into human mitochondria. *J Biol Chem*. 2010;285:30792-803.
3. Matsuura K, Honda A, Nagai T, Fukushima N, Iwanaga K, Tokunaga M, Shimizu T, Okano T, Kasanuki H, Hagiwara N and Komuro I. Transplantation of cardiac progenitor cells ameliorates cardiac dysfunction after myocardial infarction in mice. *The Journal of clinical investigation*. 2009;119:2204-17.
4. Abe J, Yamada Y and Harashima H. Validation of a Strategy for Cancer Therapy: Delivering Aminoglycoside Drugs to Mitochondria in HeLa Cells. *J Pharm Sci*. 2016;105:734-740.
