# Supplementary material for: Transplantation of MITO cells, mitochondria activated cardiac progenitor cells, to the ischemic myocardium of mouse enhances the therapeutic effect
Source: Sci Rep. 2022 Mar 22;12:4344. doi: 10.1038/s41598-022-08583-5 (PMC8941106; doi:10.1038/s41598-022-08583-5)
Supplement: Supplementary file 2 — Supplementary Information 2. [file 41598_2022_8583_MOESM2_ESM.pptx]

## Slide 1
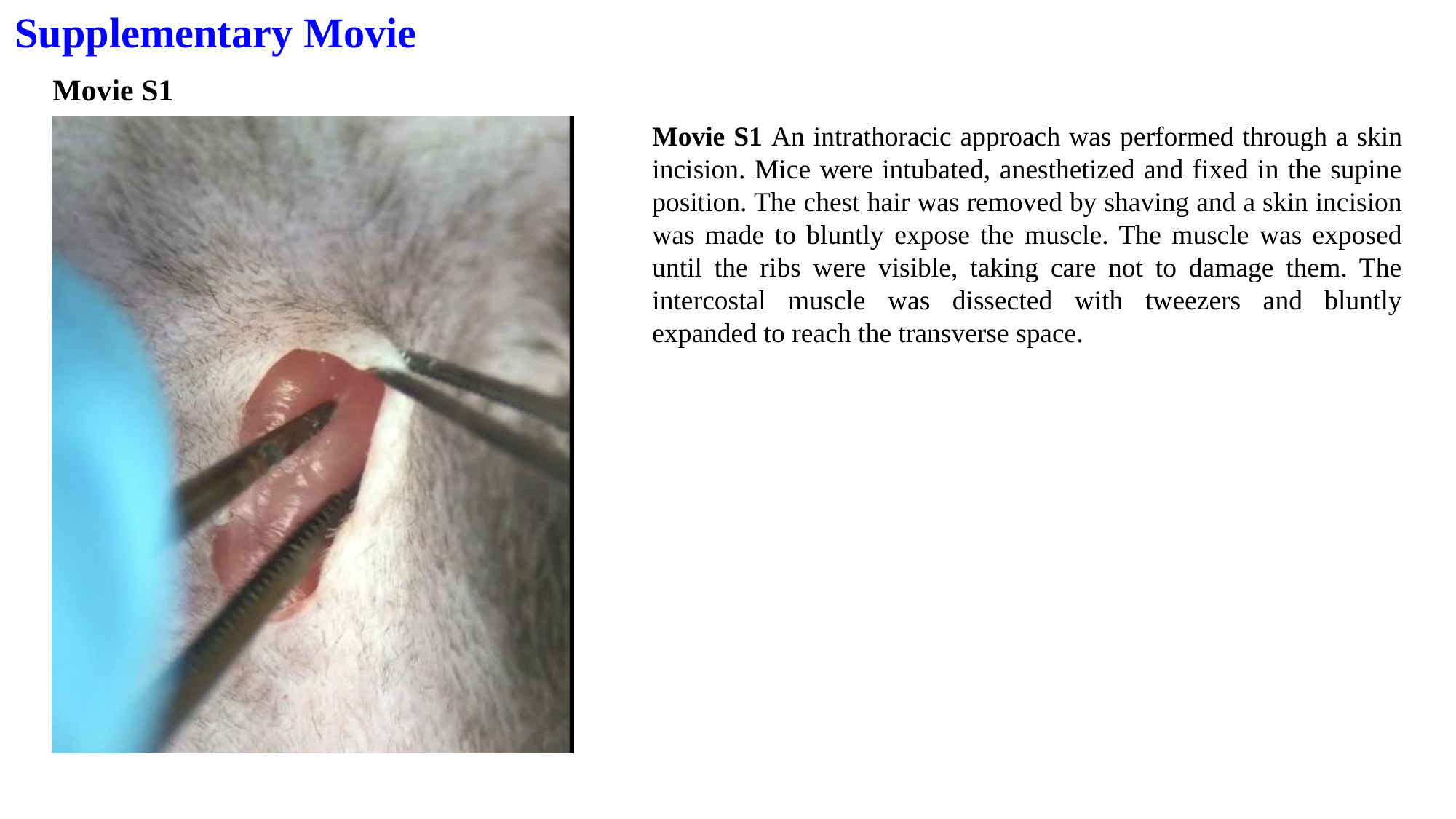

Supplementary Movie
Movie S1
Movie S1 An intrathoracic approach was performed through a skin incision. Mice were intubated, anesthetized and fixed in the supine position. The chest hair was removed by shaving and a skin incision was made to bluntly expose the muscle. The muscle was exposed until the ribs were visible, taking care not to damage them. The intercostal muscle was dissected with tweezers and bluntly expanded to reach the transverse space.

## Slide 2
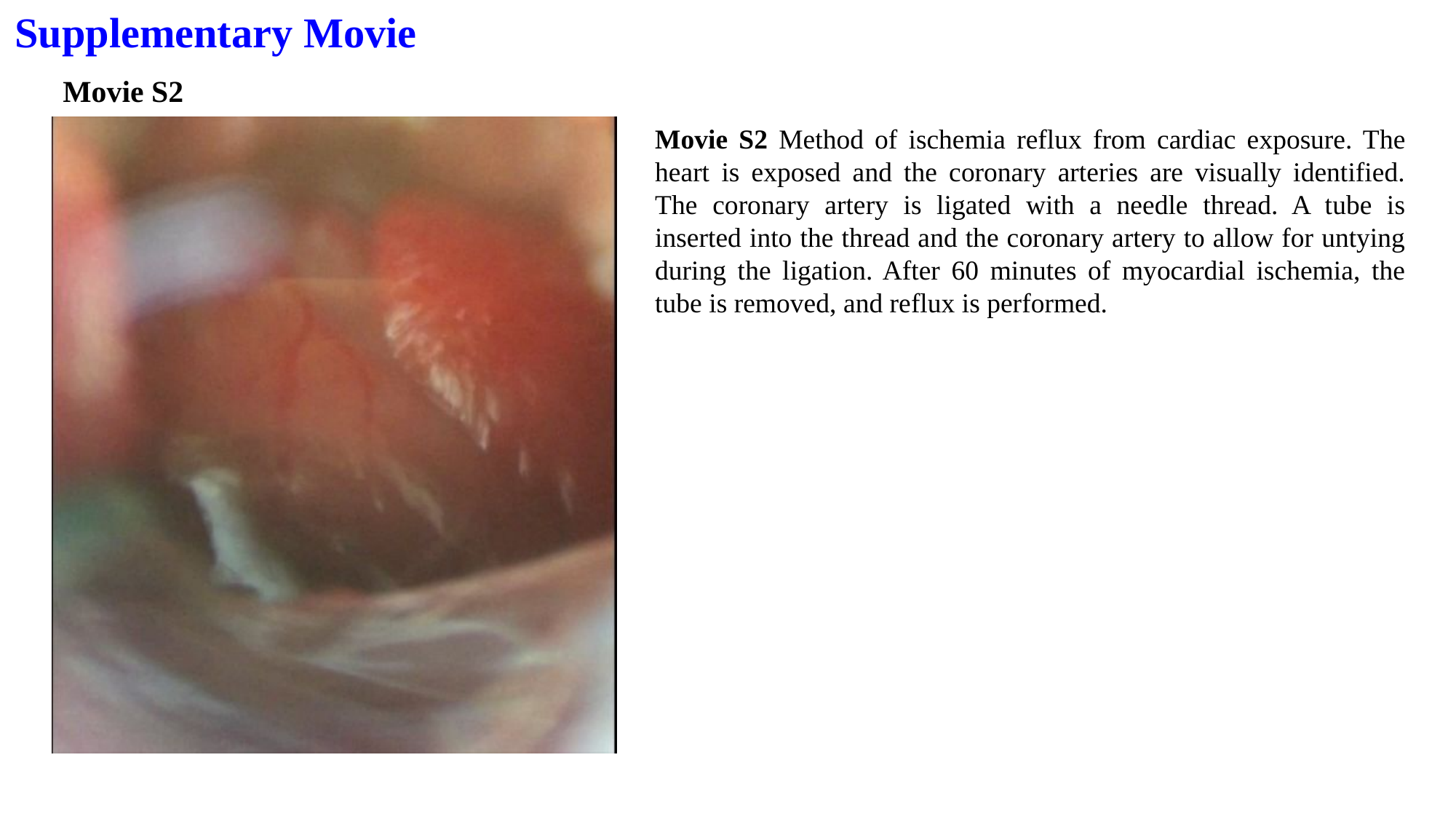

Supplementary Movie
Movie S2
Movie S2 Method of ischemia reflux from cardiac exposure. The heart is exposed and the coronary arteries are visually identified. The coronary artery is ligated with a needle thread. A tube is inserted into the thread and the coronary artery to allow for untying during the ligation. After 60 minutes of myocardial ischemia, the tube is removed, and reflux is performed.
